# User-Centered Design of a Digitally Enabled Care Pathway in a Large Health System: Qualitative Interview Study

**DOI:** 10.2196/42768

**Published:** 2023-07-26

**Authors:** Maggie McCue, Rasha Khatib, Christopher Kabir, Chris Blair, Ben Fehnert, James King, Alexander Spalding, Lara Zaki, Lambros Chrones, Anit Roy, David E Kemp

**Affiliations:** 1 Takeda Pharmaceuticals U.S.A., Inc. Lexington, MA United States; 2 Advocate Aurora Research Institute Advocate Health Care Downers Grove, IL United States; 3 Cognition Kit Cambridge United Kingdom; 4 Ctrl Group / Fora Health London United Kingdom; 5 Advocate Aurora Health Downers Grove, IL United States

**Keywords:** depression, major depressive disorder, depression management, patient engagement, user-centered design, mobile app, digital platform, qualitative research, shared decision-making, measurement-based care, mobile phone

## Abstract

**Background:**

Major depressive disorder (MDD) is a leading cause of disability worldwide. Management of chronic conditions such as MDD can be improved by enhanced patient engagement, measurement-based care (MBC), and shared decision-making (SDM). A user-centered design approach can improve the understanding of the patient journey and care team workflows and thus aid the development of digital health care innovations optimized for the needs of patients living with MDD and their primary care teams.

**Objective:**

This study aims to use qualitative research methods for the user-centered design of a digitally enabled MDD care platform, *Pathway* *Platform*, intended to enhance patient engagement, MBC, and SDM.

**Methods:**

Insights were gathered through 2 stages of qualitative interviews by a study team with expertise in qualitative research and user-centered design methods. Thematic analysis was used to generate an overarching understanding of a set of shared experiences, thoughts, or behaviors across a broad qualitative data set, including transcripts of interviews, to allow both inductive and deductive insights to emerge. Thematic analysis of interviews was supported by Dedoose (SocioCultural Research Consultants, LLC), a qualitative data analysis software tool that enables systematized coding. Findings and insights were presented based on code frequency, salience, and relevance to the research project.

**Results:**

In stage 1, interviews were conducted with 20 patients living with MDD and 15 health care providers from September 2018 to January 2019 to understand the experiences with and perceptions about the initial functionality of the *Pathway app* while also exploring the perceptions about potential additional features and functionality. Feedback about care team workflows and treatment approaches was collected in stage-2 interviews with 36 health care providers at 8 primary care sites. Inductive and deductive thematic analyses revealed several themes related to app functionality, patient-provider engagement, workflow integration, and patient education. Both patients and their care teams perceived the remote tracking of patient-reported outcomes via digital tools to be clinically useful and reliable and to promote MBC and SDM. However, there was emphasis on the need to enhance the flow of real-time data shared with the care team, improve trend visualizations, and integrate the data within the existing clinical workflow and educational programs for patients and their care teams. User feedback was incorporated into the iterative development of the *Pathway app*.

**Conclusions:**

Ongoing communication with patients living with MDD and their care teams provided an opportunity for user-centric developmental iterations of the *Pathway Platform.* Key insights led to further development of the patient-facing and care team–facing visit preparation features, collaborative goal-setting and goal-tracking features, patient-reported outcome summaries, and trend visualizations. The result is an enhanced digital platform with the potential to improve treatment outcomes and provide patients living with MDD additional support throughout their treatment journey.

## Introduction

### Background

Depression is a leading cause of disability worldwide, affecting nearly 300 million people [1,2]. Major depressive disorder (MDD) is a growing problem in the United States, with the total number of US adults with MDD increasing by 12.9%, from 15.5 million to 17.5 million between 2010 and 2018, and it is associated with a significant economic burden [3]. Primary care centers are the largest mental health service providers for people living with MDD, with up to two-thirds of visits to health care providers (HCPs) for depression occurring in a primary care setting [3-6]. Time constraints and the need to frequently manage multiple conditions during a single visit to a primary care setting can make it difficult for HCPs to fully engage with patients when it comes to their treatment for MDD [4]. Frequent communication and engagement between people living with MDD and their care teams may improve therapeutic outcomes, especially for chronic conditions such as MDD [4].

### Measurement-Based Care

The American Psychiatric Association clinical guidelines recommend measurement-based care (MBC) for treating depression. MBC includes the routine use of standardized outcome measures to assess changes in depression symptoms, level of functioning, and quality of life across the treatment course [7]. MBC for MDD is effective because it allows the primary care provider to quantify clinical outcomes. This provides guidance for timely treatment modifications that may better meet the needs of the person being treated [8]. Furthermore, treatment decisions based on MBC give people living with MDD a better understanding of how their condition is changing over time and therefore potentially empowers them in terms of their own care [8]. In addition, compared with usual care, MBC in the management of depression has been shown to improve treatment adherence, thereby leading to improvements in clinical outcomes [8,9]. Despite the demonstrated benefits of MBC in improving treatment outcomes and patient engagement, adoption of MBC in routine clinical practice has been slow, with only 20% of HCPs using it in their practice [8]. Increased consultation times are often cited as a barrier to MBC implementation.

### Shared Decision-Making

Along with MBC, another critical factor shown to improve treatment outcomes is the involvement of people living with MDD in the decision-making processes of their treatment journey [10]. Several studies have found that people experiencing a mental illness want to play a large role in the treatment decision-making process [10]. A shared decision-making (SDM) model of interaction can foster patient-provider engagement by empowering patients to play a great role in the decision-making process, thus creating an opportunity for them to have their voices, beliefs, values, goals, experiences, and preferences reflected in the treatment planning and monitoring process. This, in turn, can help increase treatment satisfaction and overall treatment adherence [10].

Among people receiving treatment for mental health disorders (including depression), increased adherence to treatments (including both psychopharmaceutical and psychotherapeutic) has been reported when they recognize that their treatments reflect their unique needs and preferences. A strong alignment between the treatment goals of the person living with depression and their treatment provider is another critical factor shown to be important in promoting adherence [10,11]. Several studies have reported misalignment between what patients and their treatment providers consider to be the most important treatment goal [10]. Thus, involvement in SDM is associated with a high probability of receiving quality care and improvement in symptoms [12] through increased adherence to drug treatment [10].

### Digital Tool Development in MDD

Digital tool development that enhances patient engagement, MBC, and SDM has the potential to improve treatment outcomes in MDD [13]. Digital communications and information technologies have previously been shown to improve health care delivery by improving communication between providers, and by decreasing the need for face-to-face appointments, thus helping to alleviate the workload of HCPs [14,15]. With rapid advances and the adoption of smartphone technology, mobile health apps have generated interest from both the public and medical communities [16]. For the HCP, digital technology platforms, such as mobile apps, can offer low-cost interventions to monitor and improve services for patient populations that are difficult to retain during treatment [15]. A mobile app could help save time because people living with MDD could engage with symptom assessments (such as the 9-item Patient Health Questionnaire [PHQ-9]) and other clinical instruments outside their visit, whereas HCPs would only need to review these results instead of administering the instrument during the visit itself [8]. A mobile app could therefore promote MBC by allowing patients to remotely engage with validated quantitative measures of assessments, which can then be uploaded into their electronic health records (EHRs) for in-office visits and physician monitoring [8]. Collection of PHQ-9 results through a mobile app has been shown to be as sensitive as, or even more sensitive than, the traditional (in-person and paper-based) PHQ-9 data collection method [17]. This may be because people living with MDD might feel more comfortable revealing their symptoms in remote settings through a mobile app rather than in traditional in-person settings.

From the patient’s perspective, several studies using smartphone app–based interventions for depressive disorders have shown that depressive symptoms were reduced significantly more with smartphone apps [16,18,19] through motivating some users to consult medical professionals for diagnosis and management [20,21] and through self-observation [22,23]. Furthermore, apps dedicated to the caregivers of people living with MDD have also been shown to help caregivers in better supporting their loved ones and to destigmatize mental health care [24].

### User-Centered Design Approach

Achieving this potential of digital tools to improve treatment outcomes requires a nuanced understanding of the desirability and usability of patient-facing and care team−facing digital interfaces alongside the practical requirements for patient and care team adoption. Perceived utility and overall value of the product to care teams and privacy and confidentiality concerns are often cited by care teams as examples of barriers that can limit the adoption of these types of apps [25]. Various mobile apps are available for depression management; however, many are patient facing only and do not include a care team interface [26]. The lack of guidance or feedback from the care team has often been cited by people living with depression as a barrier to the adoption of many apps that are dedicated to mental health care [25,27,28]. Patients might, for example, perceive the lack of feedback or engagement from the care team as indicating that the information they are providing through the app is not being monitored or integrated into their care processes, thus disincentivizing their engagement with these products [29].

A user-centered design approach can improve the understanding of the patient journey (Multimedia Appendix 1) [30]. Patient insights help develop an understanding of user needs, with iterative designs and prototypes playing a key role in how these insights and needs can be unearthed [30]. However, studies describing the user-centered design approach in health-related technology remain limited [30]. Takeda, Lundbeck, and Advocate Aurora Health (AAH) partnered to cocreate a digitally enabled care experience with users (patients living with MDD and care teams), software developers, and health-technology product development specialists (Ctrl Group and Fora Health) [31]. Specifically, a digital mobile patient interface, the *Pathway App*, was designed with a conversational interface and tested via a pilot feasibility study with 40 patients living with MDD [31]. The study showed a trend toward high patient activation and patient-provider engagement for people who used the app in addition to usual care compared with those who were assigned to usual care only [31]. Building on these results, a new iteration of the app was created and incorporated into a new digital platform, *Pathway Platform*, which includes the *Pathway App*; EHR-integrated, real-time, patient-level data sharing; and educational programming that is both care team and patient facing. This was guided by a more comprehensive understanding of care team workflows and patient and care team insights [32]. In this paper, we describe how user-centered design was applied to develop a digitally enabled MDD care platform that is optimized for the needs of patients living with MDD and their primary care teams.

## Methods

### Overview

This report describes the qualitative research undertaken to understand, iterate, and integrate *Pathway Platform* into primary care in the AAH system within and around Chicago, Illinois. In stage 1, interviews were conducted with 20 patients with MDD who participated in the pilot feasibility study and 15 HCPs (from September 19, 2018, to January 30, 2019). The stage-1 interview sought to understand the experiences with and perceptions about the initial functionality of the *Pathway app* (Multimedia Appendix 2) while also exploring perceptions about potential additional features such as HCP visit preparation, patient education, and goal setting and tracking. Interviews with patients lasted up to 60 minutes, and interviews with HCPs lasted up to 30 minutes. Another round of follow-up feedback was collected from 36 HCPs at 8 primary care sites through stage-2 interviews to understand care team workflows in the treatment of patients with MDD, the extent to which *Pathway Platform* can help optimize care, and what support the care team will need to make *Pathway Platform* work at their respective sites. HCPs were included if they were involved in primary care and specifically managed the care of patients with MDD. The semistructured, in-person interviews were conducted by a study team with expertise in qualitative research, user-centered design methods, workflow assessment, and educational support (Ctrl Group and PRIME Education LLC). Care teams were interviewed about topics related to workflow, perceptions about and experiences with MBC and SDM, and educational needs of patients with MDD and their care team members. The interviews were conducted until observational and analytical saturation was achieved. Saturation was defined as when no new inductive themes emerged during analysis and a priori or deductive themes were exemplified in the data [33].

### Data Analysis

The qualitative data were first coded and analyzed independently by 2 Ctrl Group researchers using a set of foundational structural codes. A structural coding framework, based on the agreed-upon discussion guide from the qualitative interviews, was created for the “top-down” codes; “bottom-up” codes that organically arose from the data were also used. The top-down and bottom-up coding was then refined collaboratively as the researchers progressed through the data set by, for example, splitting preexisting codes into more specific subcodes and combining existing subcodes where appropriate. Once refined, the codes enabled thematic analysis and identification of recurring themes across participants’ accounts and perceptions. Guided by prior research findings [34-36], we adopted thematic analysis to generate an overarching understanding of shared experiences, thoughts, and behaviors across a broad, qualitative data set, to allow both inductive and deductive insights to emerge. This was particularly important because our study aimed to validate preexisting product features (through deductive reasoning) while also allowing respondents to provide suggestions that can inform the development of completely new features (inductive reasoning). This type of mixed reasoning would have been very difficult to achieve if the participants had only been surveyed about their thoughts around specific product features. The flexibility allowed by the thematic analyses was also important because it enabled engagement with both personal accounts of patients’ experiences and understandings and broad social constructs (eg, “workflow efficiency” or “medication adherence”) in slightly different social contexts (ie, different primary care environments) in the same research [36].

Thematic analysis of interviews was supported by Dedoose (SocioCultural Research Consultants, LLC), a qualitative data analysis software tool that enables systematized coding. Audio recordings from the interviews were uploaded to the web-based transcription service, Rev. Transcripts were uploaded to Dedoose [37], the top-down codes created during the coding framework phase were applied to appropriate excerpts for each transcript, and new bottom-up codes were created and applied as they arose over the course of the analysis. High-level analyses were conducted by reviewing the codes that had been applied to the interview transcripts. This was done to identify key and recurring themes from the insight-gathering stage. Interpretive insights were formulated through subsequent in-depth analyses. Findings and insights were presented based on code frequency, salience, and relevance to the research project.

The Standards for Reporting Qualitative Research recommendations were followed in the reporting of the study [38].

### Ethics Approval

Ethics approval from the AAH institutional review board was obtained by making amendments to the existing Advocate Pathway study protocol (approval number: AHC-6680-75000249).

## Results

### Participant Characteristics

A total of 37 patients living with MDD completed the 18-week primary follow-up period in the pilot feasibility study—19% (n=7) were Black and 38% (n=14) were Hispanic [31]. Overall, 54% (20/37) of patients from the pilot feasibility study (9/18, 50% from the *Pathway App* arm and 11/19, 58% from the usual care arm) participated in the qualitative interviews in stage 1. In addition, 15 HCPs also participated in the stage-1 interviews. Overall, 53% (8/15) of HCPs in the group were previously involved in the pilot study, 25% (2/8) of whom were assigned to the *Pathway* *App* arm and had used the app to derive patient reports. A total of 36 HCPs from primary care medicine with experience in managing people with depression participated in the stage-2 interviews (Multimedia Appendix 3). These HCPs were sampled for diversity of roles both within and across sites, and it was found that 33% (12/36) were physicians, followed by certified medical assistants (8/36, 22%), registered nurses (6/36, 17%), and licensed practical or advanced practice nurses (6/36, 17%).

### Users’ Experiences With and Perceptions of the Pathway App

#### Overview

Thematic analysis identified major themes to describe the users’ experiences with and perceptions about the *Pathway App*, which included functionality, support, and patient-provider engagement, along with subthemes such as ease of use, utility, reliability, motivation, reducing burden, communication, understanding, and shared vision (Tables 1 and 2).

**Table 1:** Experiences with and perceptions of the *Pathway App* among patients living with major depressive disorder and health care providers.

Themes, subthemes, and the *Pathway App* features				Participant quotes
**Functionality**				
	**Ease of use**			
		Side effects	“It’s really easy to see how your meds are affecting you...” (Patient; app)	
		Mood	“It was easy to use because it had good options...it’s hard to pinpoint how you’re feeling on a numbers scale...easier when you’re explaining it.” (Patient; app)	
	**Utility**			
		Medication tracking	“...very helpful because if you’re really busy you get notifications that remind you to take it. I used it consistently. I forget a lot and that thing would pop up...everyone needs a reminder especially if you’re on new meds or daily meds.” (Patient; app)	
		Side effects	“A lot of mine were on there and it was super easy to use...as time went on I realized I wasn’t having them anymore.” (Patient; app) “It’s great data to see and it’s nice that you can track that, especially with the medication in compliance and the side effects.” (RN^a^)	
		Mood	“...pretty useful because it would make me realize how well or badly I was doing...I started meds in July and didn’t feel them until September, October...” (Patient; app)	
	**Reliability**			
		Mood	“I think this is beneficial, especially if you see the data and notice, for example, on Saturday the patient seems to be down—what’s happening on this day from a psychological perspective...if they’re always down then maybe you need to increase medication...this gives me objective data.” (HCP^b^; study) “We can know definitively what's going on with the medication and not rely on the patient to give us a history, because they’re more inclined to be honest with their phone on a day-to-day basis and not give us generalities when they come in a month later...This definitely would give us a better picture. More accurate.” (LPN^c^)	
		Medication tracking	“Patients like follow-up and reminders. Medication adherence data can be difficult to get reliably so useful in that sense...” (HCP; nonstudy)	
**Support**				
	**Motivation**			
		Medication	“It actually makes you feel like you’re talking to somebody...It lifted my spirits at the end of the day and it’s easy to navigate...very useful because it will make me think...” (Patient; app)	
		Side effects	“Sometimes I was looking forward to tracking how I was feeling to keep track of side effects...I really had not been on this type of medication before...so this was just to learn which side effects I was actually having and to tell the difference between the medication to see which would give me least side effects...so it just helped me figure out maybe this is not the best type to be taking...” (Patient; app)	
		In-app report	“I like the visual—it helps you remember you need to take your meds every day...it’s very useful to see side by side and broken down by week.” (Patient; usual care) “Especially for young people it’s important to try and remind them. The look is nice...[it has a] pleasant appearance...and provides feedback which is helpful...it looks encouraging too, there’s positive reinforcement...” (HCP; nonstudy)	
		Goal setting and tracking	“I think it gives you something to look forward to. It helps when you’re dealing with depression...gives you a purpose.” (Patient; app) “I like to be able to say that I almost got there or I made a little progress...It would help motivate you...” (Patient; usual care) “‘Almost got there’ is good language, it’s supportive...It’s really a form of motivational interviewing and it allows you to go a little further with more information behind you...” (HCP; nonstudy)	
		Patient education	“This helps you focus back on yourself. I’m jealous...I want to use it...I was already thinking of this as an addition.” (Patient; app) “I love this, it feels very enticing. It would definitely keep me engaged...[it’s] peaceful and motivating.” (Patient; app)	
	**Reducing burden**			
		HCP visit preparation	“As patients we always write down all our questions, but the doctor only has so much time—a lot of time is just the provider asking a lot of questions, trying to get down to the main thing—this will cut time on the talking...[It’s] easier for shy patients to point out their concerns to their provider. I think doing the questions before the appointment, will actually help” (Patient; app) “I really like that because I’m seeing it almost as a time saver...I’m trying to tease out information and giving them the opportunity to process things ahead of time you’ve already got that foundation...I feel like that would be useful to me and the patient...” (HCP; study)	
		Side effects	“...This was helpful especially when you go to the doc because you don’t have to try and remember three or four weeks ago...It’s actually nice to be able to tap in side effects without having to go to the doc’s for three hours just to tell them you have dry mouth or fatigue.” (Patient; app)	
		Patient education	“I really like this because I don’t take the time to do this with the patient. I think patients would engage with it...Aside from treatment the whole behavioral therapy component is key. I think it would make my life a lot easier because it takes a lot of time out of clinical practice.” (HCP; nonstudy)	

^a^RN: registered nurse.

^b^HCP: health care provider.

^c^LPN: licensed practical nurse.

**Table 2:** *Pathway App* and its effect on patient-provider engagement.

Themes, subthemes, and the *Pathway App* features		Participant quotes	
**Communication**			
	Goal setting and tracking		“It helps make it more intimate where they’re not just trying to medicate you...it’s outside of just looking at you as a patient...it makes it more personal...” (Patient; app) “I love it...this is so exciting for me! This is what I try to do manually but this would be so much more effective...you deepen the conversation.” (HCP^a^; nonstudy) “It’s important for the physician to have that information. It helps to hold the patient accountable...to look in black and white and have an honest conversation with the doctor...” (Patient; usual care)
	Side effects		“You can say to someone let’s see what happens after a month. It makes it easier to attribute side effects to meds or other things...which supports the conversation with the patient.” (HCP; nonstudy)
	Patient education		“Going up a hill is a good visual—things seem hard but if you pick a goal and pick away at it...I like the language of getting back to yourself because you’re not yourself and you don’t feel yourself...This would be useful to me...I would want to know the content so I could have conversations and would know where to focus...this would balance the visit out a little bit...I love it.” (HCP; nonstudy)
**Understanding**			
	For all tracking features		“Great idea...you don’t have to remember every single feeling, every single side effect...if they see an issue during that time they can go right to that point and not waste 35 minutes trying to figure out how you were possibly feeling and trying to remember way back to that day...they just have it right there...and they can say OK, you were feeling this particular way, how are you now?” (Patient; app) “I would 100% use this with my patients...[This is] especially good for someone starting or changing meds...now you could know how meds affect sleep, energy...I would want time to digest this so would be good to receive prior to any appointment and to receive on an ongoing basis.” (HCP; nonstudy) “There’s a very good indication over time of how someone’s feeling instead of just at this visit when they walk in and [are having] a bad day or a good day.” (RN^b^)
**Shared decision-making**			
	For all tracking features		“I think it would engage the patient more. They could see that I’m getting the data from them and taking it seriously, and that someone is interested in their condition and is monitoring their condition, so I think it would be good for the patient.” (MD^c^) “If I’m seeing it before the patient came in, the PHQ-9 is already done so I don’t have to ask those questions which saves me a little bit of time, and then I would get into ‘looks like you’re still doing not as well as we’d like to,’ so I would go back into shared decision-making.” (MD)

^a^HCP: health care provider.

^b^RN: registered nurse.

^c^MD: doctor of medicine.

#### Functionality

Functionality was a major theme that emerged from the interviews, with 3 associated subthemes—ease of use, utility, and reliability. Patients living with MDD and HCPs emphasized ease of use and overall value as 2 of the most beneficial app features. Participants appreciated the simplicity of the design, which made it easy to understand and use the app effectively, and many reported that the app provided clinically meaningful information, such as tracking of symptoms, mood, and medication adherence, which helped them manage their symptoms more effectively. HCPs viewed mood, medication, and side effect tracking to be clinically valuable, owing in part to the continuous and direct input from the patient.

#### Support

Support was an important theme that emerged, which could be split into 2 subthemes—motivation and burden reduction. Patients living with MDD reported that the goal-setting and goal-tracking features provided emotional support and motivation through regular reminders and encouragement, which helped them stay engaged in their treatment.

Similarly, HCP visit preparation was noted as very useful by patients living with MDD. Many reported that the ability to prepare for HCP visits using the *Pathway App* reduced their anxiety and improved the ease and accuracy of communication with their HCP.

HCPs felt that the visit preparation feature reduced their administrative burden and improved the efficiency and effectiveness of appointments. Similarly, HCPs stated that patient education features reduced the education burden on them, providing a constructive focus for patients living with MDD and improving patient-provider interactions.

#### Patient-Provider Engagement

Patients living with MDD reported that the app helped to improve their interactions with their HCP by providing easy-to-use tools for tracking symptoms and progress. They also appreciated the ability to share their data with their HCP, which they said helped to facilitate better communication and collaboration. Patients and HCPs found that the goal-setting and goal-tracking features of the app supported clinical conversations while also helping patients focus on their treatment goals. HCPs reported that patients may remain engaged in their care by knowing that their input and concerns are being taken seriously, thereby promoting SDM.

Both patients living with MDD and HCPs expressed positive feedback about the patient education feature of the *Pathway App*. They found the feature to be visually appealing and engaging and deemed the content to be suitable in length and depth. Furthermore, HCPs reported that the education feature would facilitate productive conversations with patients.

### Concerns

Concerns about the *Pathway App* were that it was difficult to use and had a lack of interactivity, with subthemes such as being confusing, repetitive, and time consuming and lacking workflow integration (Table 3).

The initial well-being tracker, a visual analog scale from 0 to 100, received the most critical feedback from patients living with MDD, as many of them found the construct and scale to be unnecessarily complicated. HCPs also expressed skepticism about the clinical utility of the well-being tracker feature and had concerns about the ease with which they could interpret the responses. Both HCPs and patients suggested changes to include a simple response format and visual indicators to support interpretation. The cognition 2-back feature also generated negative reviews, with both patients and HCPs expressing their frustration, as many found it to be confusing and anxiety inducing, and they expressed skepticism about the usefulness and interpretation of data. Participants also expressed frustration about the daily repetition of certain questions and expressed concerns about disengagement.

**Table 3:** Concerns about and recommendations for the *Pathway App* by patients living with major depressive disorder and health care providers.

Categories, themes, subthemes, and the *Pathway App* features					Participant quotes
**Concerns**					
	**Cumbersome to use**				
		**Time consuming**			
			Pathway report	“This is helpful but I would really love a 1-page analysis report because I don’t have a lot of time...” (HCP^a^; nonstudy)	
		**Confusing**			
			Well-being tracker	“I didn’t find it as useful as the others...I just left it at 50...I don’t know why. It was just a little confusing.” (Patient; app) “I did like it but it’s a little too complicated...what is the point of saying I feel like 86 or 62 today? A 1-10 scale would have been easier to use.” (Patient; app) “That’s a very large range. What’s good? What’s bad? Is 80 good? Sometimes giving too large a range is too much for patients to think about. What does that really mean for me?” (HCP; study)	
			Cognition 2-back	“I got aggravated. I didn’t understand the whole concept and I failed. It never explained the purpose. I have no idea what it was trying to do...it was so vague, it just said ‘do you want to start?’ Maybe I felt I failed because of the scores...I didn’t get a lot of greens...I stopped doing them...I didn’t understand why or what I was doing...” (Patient; app) “The 2-back thing needs to go or explain it more or more clarification—I wouldn’t take it away completely; it just needs more meaning behind it.” (Patient; app)	
		**Repetitive and redundant**			
			Side effects	“...Does it have to be on a daily basis or could it be weekly? I could see it being redundant...” (HCP; study) “[I] would caution against patients feeling overwhelmed by the options if they saw them all at the same time.” (HCP; study)	
			Mood	“These questions are really good but every day? I was over it by the third day in a row. If I’m depressed, I don’t want to be reminded...If I’m having a good day, I don’t want to trigger it...[so] I would ignore it...it’s a lot...reminding myself I feel so low all the time...” (Patient; app) “...It seems like a lot to ask about well-being and mood...I’m less concerned about daily stuff and more concerned with weekly trends...my intuition is that you don’t need this every day and I would worry about overloading the patient.” (HCP; study)	
	**Lack of interactivity**				
		In-app report			“I think you should be able to put in your time frame and ask the app to show me my progress over this amount of time. Make it a fun thing—a metric or a tab where people can see progress over time in a fun way...People are motivated by progress, right?” (Patient; usual care)
**Recommendations**					
	**Workflow integration**				
		Pathway report			“...a 1-page report regardless of the period of time since the last appointment that could be faxed or e-faxed back into the EMR^b^...” (HCP; nonstudy)
		HCP visit preparation			“I might not be able to look at it before the visit. I can look at it quickly with the patient there as long as it’s simple for me to read, I can scan and we can have a conversation...ideally this would be linked to EMR...integration would help, it would give me more time to talk to them.” (HCP; study) “The sharing it with your physician part makes me raise an eyebrow because that has to be very seamlessly integrated in a way I’m not sure is possible currently...so unless the progress concerns are uploadable into the EMR and integrated into a physician’s workflow...it’s going to be really difficult to follow up on the fact that you told my patient that I have this information...if that [EMR integration] is possible that would be fantastic because it takes care of some of my documentation too because it’s as if I’ve asked these questions even though I haven’t.” (HCP; study)
		Goal setting and tracking			“If I’m entering anything in my computer, I don’t want to retype, I want it to automatically link. I don’t want to spend any more time on the EMR. I feel like I’m on it 24 hours a day already. If I’m typing in goals, I want it in my progress notes.” (HCP; study)

	**Increase interactivity**				
		Patient education			“Is it going to elaborate more? Like articles and things you could work on or...informational things like why you might be feeling the way that you’re feeling...like help you, make you think a little, try that thing, see if that helps me or even if it was something small like today—have you thought of going outside and taking a deep breath in nature...or like have you sat down and meditated for 5 minutes? It doesn’t have to be a lot, but little things to guide you...” (Patient; app) “One thing I haven’t seen is little videos or motivational stories or patient stories or different things to engage patients...or other vendors that offer CBT^c^...or Reddit group support or offers to join support groups...” (HCP; nonstudy)
	**Explanation and visualization**				
		Cognition 2-back			“The 2-back thing needs to go or explain it more or more clarification—I wouldn’t take it away completely; it just needs more meaning behind it.” (Patient; app)
		Well-being tracker			“Anything you can make simpler you should make simpler.” (HCP; study) “0-100 is a lot of in between...I don’t know how I would rate myself. I just think of the smiling faces [in the hospital]...they ask you on a pain scale of 0-10.” (Patient; usual care)

^a^HCP: health care provider.

^b^EMR: electronic medical record.

^c^CBT: cognitive behavioral therapy.

### Recommendations

For the well-being tracker, participants suggested a simple response format and the inclusion of visual indicators to support interpretation (Table 3). A better explanation and provision of an alternative cognitive exercise were suggested by participants for the cognition 2-back feature. Providing examples of goals and assisting patients living with MDD with setting their own goals independently of their care teams were among the HCP-suggested changes to the goal-setting feature. Both HCPs and patients suggested changes to goal tracking, such as options to set goal reminders, record goal progress, and provide rewards to motivate goal attainment.

Many of the HCP-suggested changes included an emphasis on the value of EHR integration for easy access and to save time. They suggested that features such as HCP visit preparation, goal setting and tracking, and the *Pathway* report would benefit from integration with the EHR to be more useful and effective for HCPs. In addition to workflow integration, HCP-suggested changes included increasing the interactivity and variety of the given content for patient education, while also making the value and purpose of the educational materials clear.

### Optimization of the Integration of the Pathway App Into a Primary Care Clinical Workflow

#### Overview

Care team members made several recommendations regarding how to facilitate the integration of the *Pathway App* into clinical workflows. These included product-specific recommendations and suggested changes to existing workflow scopes. Key themes that emerged were the importance of MBC, SDM, educational needs for the care team, and patient education (Table 4).

**Table 4:** User perceptions and experiences regarding the integration of the *Pathway App* into a primary care clinical workflow.

Categories, themes, and subthemes				Participant quotes
**Perceptions**				
	**Importance of MBC^a^**			
		PROs^b^	“It definitely gives you a tracking method. You’re able to really see over a period of time how the patient’s doing, which is key, especially for a depressed patient. You may only see them once every 6 months, which again, you don’t get the exact data that you need during that period...you’re really relying that the patient will follow up regularly...If not, at least the app is giving you an idea of what’s going on.” (RN^c^) “It just means making me much more aware of that because I don’t always have the time to dig into this with my patients. I think it makes me a better PA^d^ overall if I’m able to touch on these things. And sometimes it’s just nice to have a reminder right there in front of you while you’re talking to your patients.” (PA) “I think it’s nice to actually see it, especially now that you’re tracking it over a period of time and you can see how it’s increasing, decreasing things of that nature. So I think that’s a great method to have it like that. And it’s not too much information, it’s just enough data that shows me where they’re going.” (RN) “It’s really just making sure that they’re progressing or their symptoms are improving with their medication. Again, some patients miss visits even if they’re supposed to follow every 2 months and then they don’t make it to that 2-month visit. If Pathway can communicate with that patient and I can see the score, I’ll be able to correlate that with the effectiveness of the medication.” (MD^e^)	
	**Shared decision-making**			
		Goal setting	“You’re able to track if the patients are really adhering to the goals that you set. And it’s more frequent versus me asking them once or twice in the office. This is a more frequent check...” (RN) “I think it’s really helpful. It’s going to definitely engage the patient with their care and simplify, not just send them on their way. ‘This is what I want you to do.’ It’s tangible, it’s on your phone, there’s no question as to what was asked or talked about at the visit. It’s right there. I think it will be great for patients.” (LPN^f^)	
		Side effects	“Well, I always ask them what side effects they’re having, but I guess when they come in, I can reinforce they’re side effects, or is it their illness itself based on what I’m seeing here. So...the insomnia, well, I’ll ask them, how’s the medication working, and they might say it’s working well, but they might not say that they’re having insomnia, so I can get that from here. So that would be helpful.” (MD) “I would go back into the whole shared decision-making...‘We tried sertraline before. It looked like you did have some side effects, although they went away. How would you feel about going up on the dose of the medication because I’d like to try to tap out before I switch?’” (MD)	
**Concerns**				
	**Overwhelming**			
		Pathway data	“As a physician I worry...with this information, what if I miss something? Because there’s so much information; if I don’t know where to find it, how to use it...especially where to find it...In a timely, effective, efficient way, I’m afraid that, what if I miss something that is crucial for this patient?” (MD)	
	**Educational support**			
		Cognition tests	“Maybe there was some training for the provider so it’s like actually this tracks really well with how their cognitive performance is doing with depression. Then [I’d say] ‘Okay, you got me. I'm in.’” (MD)	
		PROs	“I don’t know enough about this WHO^g^ or the PDQ-D-5^h^, what these numbers mean. And if they’re getting better, or worse. I’m just not familiar with those. And then the same thing with these 2. I guess I just don’t know enough about these 2 scores.” (APN^i^)	
**Recommendations**				
	**Patient education**			
		Educational resources for patients	“I’ll give them handouts and such that I find on say, UpToDate, or embedded in our EMR^j^.” (PA) “I think we’re always weary, but I think more knowledge is better generally with patients, rather than them being surprised. [...] I would say drugs, actions, and side effects would be useful.” (MD)	

^a^MBC: measurement-based care.

^b^PRO: patient-reported outcome.

^c^RN: registered nurse.

^d^PA: physician assistant.

^e^MD: doctor of medicine.

^f^LPN: licensed practical nurse.

^g^WHO: World Health Organization.

^h^PDQ-D-5: Perceived Deficits Questionnaire–Depression.

^i^APN: advanced practice nurse.

^j^EMR: electronic medical record.

#### Importance of MBC

Although many primary care professionals within Advocate Aurora primary care perceived MBC, collaborative care, and SDM as important, various barriers limited their inclusion in day-to-day clinical workflows. Care teams agreed that *Pathway* administration of the PHQ-9 builds on and improves current clinical practices. HCPs viewed the PHQ-9 as a key component of current clinical practice; therefore, its continuous use to track patients’ treatment progress outside visits, as enabled by *Pathway Platform*, was perceived as clinically useful.

#### Shared Decision-Making

Tracking collaboratively devised goals was perceived as useful for the care team, with many also stating that this would help engage people living with MDD in their own care. HCPs reported that the presentation of patient-reported outcome (PRO) trajectories for medication adherence, PHQ-9 scores, and side effects provided a clinically useful view of how each patient’s condition has changed over time, which, in turn, supports better clinical decision-making. HCPs suggested including more information about patient care in the *Pathway*-EHR interface, such as medication refill data for comparison with reported adherence.

#### Workflow Integration and Data Visibility

Interviews also highlighted the importance of understanding care team needs, such as interpreting PRO measures and trend visualizations, to ensure that *Pathway Platform* can support care team workflows. Data about medication adherence, PHQ-9 scores, and side effects were perceived to be the most clinically important PROs. However, there were also concerns that *Pathway* data would be overwhelming and may lead to key data being missed.

#### Educational Needs

Many expressed low familiarity and desire for education regarding PROs other than PHQ-9 and clinical use of cognitive tests. In addition to their own education, the care team members also expressed a desire for patient-directed educational materials. Many care team members spoke about patients living with MDD being provided with educational handouts at their respective practices (including handouts printed from web-based searches, those that have been externally printed, and those taken from web-based medical reference platforms and the EHR). Many care team members stated that, from the list of educational topics (which included understanding one’s diagnosis, treatment options, side effect management, goal setting, and patient engagement), they were most interested in accessing materials about helping patients understand their diagnosis and their available treatment options. Care team members also thought that patients would benefit from educational resources about the types of local behavioral health resources that are available to them and information about what type of role they can play in their depression care and how often they should follow-up with their physician.

## Discussion

### Principal Findings

Qualitative research and thematic analyses conducted in this study allowed us to capture the user experiences and perceptions of patients living with MDD and their care teams. These results can guide researchers and app developers in designing effective digital health interventions that will be readily accepted by their intended end users [30].

Most of the currently available apps developed for depression management have only been assessed for effectiveness in a research setting and have not been integrated within clinical workflows; this has resulted in a lack of adoption by care teams and broad health care systems [26]. Incorporating the voices of people living with MDD and their care teams into the product development process aligns with the broad paradigm shift toward patient-focused decision-making and SDM between patients and their providers, ultimately leading to high-quality, fully informed, and preference-based treatment plans [39]. Our analyses revealed that both patients and their care teams perceived the remote tracking of PROs via digital tools to be clinically useful and reliable. Other highlights of our study included the need to enhance the flow of real-time data shared with the care team and the need to integrate within the care team workflow, including real-time sharing of the patient’s app data within the EHR. Results from our analyses also highlighted the need for care team education about MBC and SDM and about how to use the *Pathway App* to improve these processes by using features such as visit preparation and collaborative goal setting and tracking. Using the broad insights gathered from our thematic analyses, we were able to understand, iterate, and integrate a digitally enabled platform, *Pathway Platform,* into a primary care setting in the United States. The first iteration of the *Pathway App* included PRO measures related to depression, well-being, cognitive symptom tracking, medication adherence, and side effects [31]. Pilot results confirmed the feasibility of using the *Pathway App* among patients living with MDD and showed a trend in enhanced patient activation in the app arm, albeit in a small sample size [31]. Building on the results from the pilot study, *Pathway Platform* was developed to consist of 3 components: the latest iteration of the *Pathway App*; EHR-integrated, real-time, patient-level data sharing; and educational programming that is both care team and patient facing (including a web-based educational resource center that describes the utility of *Pathway Platform* to the care team through reading materials, presentations, and videos; Multimedia Appendix 4). The current version of the app prompts patients living with MDD to complete the following scales every 2 weeks: PHQ-9 [40] and Perceived Deficits Questionnaire—Depression [41] to assess depression status and subjective cognitive impairment, World Health Organization Well-being Index [42] to assess quality of life and emotional well-being, and Digit Symbol Substitution Test [43] as an objective measure of cognition to assess working memory and processing speed.

The *Pathway App* also includes a daily “evening check-in” to collect information about medication adherence and side effects. Data collected by the *Pathway App* are electronically transmitted and stored in an EHR-integrated web interface. These data are accessible to the care team and provide a longitudinal summary that may assist them in clinical decision-making and overarching depression management. Care team members can view these data either before or during the clinical visit and then use the data to collaboratively discuss future treatment decisions with the people they are treating. A web-based educational training program for primary care team members was also developed by using evidence-based medicine, building on the concepts of MBC and SDM, as they relate to depression management.

In addition, audit and feedback sessions will be conducted to benchmark performance measures, reflect on current clinical practice and improvement strategies, and set team-based action plans. Specific training sessions were conducted for the care team members about how to instruct patients to use *Pathway Platform* and how to use EHRs to view data collected via *Pathway Platform.* A training manual was also developed for patients living with MDD that describes the functionality of *Pathway Platform* and how to use and interpret their data.

*Pathway Platform* was cocreated with input from all users (people living with MDD, care teams, health system information technology personnel, and study collaborators), along with input from software developers and health-technology product development researchers, to optimize usability, utility, iteration speed, and integrated system performance and to ultimately enable nuanced care focused on SDM and MBC (Multimedia Appendix 5). Continued reassessment based on user feedback has allowed for fast iterations, optimized system performance, and sustainability [39]. This user-centric approach has, in turn, led to an enhanced digital platform to improve treatment outcomes by supporting an expanded understanding of MDD treatment, bolstering care team workflows, and providing patients with additional support throughout their treatment journey.

### Limitations and Future Directions

A limitation of our study is its small sample size of patients with MDD and their care teams. In addition, among the care teams, only 33% (12/36) were clinicians. Future studies would benefit from an even split between clinicians and nurses in the sample. Moreover, this was a single-provider network study, and the results of this study may have limited generalizability. In addition, although the design and development of *Pathway Platform* were guided by a deep understanding of care team workflows, the extent to which clinical workflows can be modified to accommodate the adoption of *Pathway Platform* will ultimately depend on the clinical team.

Furthermore, although thematic analyses offered the necessary tools for organizing, interpreting, and transforming data without the need for separate theories, the depth of our conclusions may have been increased by additional methods such as modeling and theory building [36]. However, creating an overarching or generalizable theory to explain the way people reacted to the product’s feature set as a whole [44] would have gone beyond the primary interests of the study, which were focused on understanding and analyzing specific feedback such that it could be mindfully applied to iterations of the product features. We also used a highly systematized and enumerative approach to coding and generating themes, as recommended by the guidelines [34]. In addition, we minimized interpretive inconsistencies by using a single code tree and 2 analysts to evaluate each other’s work for analytical consistency. The improved iteration of *Pathway Platform* is being evaluated in an ongoing large-scale implementation study (*Use of a Digitally Enabled App With Clinical Team Interface in the Management of Depression*; NCT04891224). The study will include up to 200 patients at 20 primary care clinics. The implementation study aims to test the scaling and integration of *Pathway Platform*, along with educational interventions, at multiple primary care sites within the AAH system, with the primary objective of determining improvement in adherence to MBC practices [32]. Results are expected to provide insights into the improvements in clinical workflows that are necessary to enhance collaborative care, depression management, clinician and patient experience, adherence to medication, patient-provider engagement, and depression outcomes in the primary care setting [32]. EHR integration and how it enables decision-making, and efficiencies with current AAH information technology platforms, such as ease of access of data in real time by the care team, will also be assessed. Together, insights from this study will allow further amendment of workflows to ensure the optimal use of *Pathway Platform* [32].

Providing effective care for MDD has become more important than ever, with the prevalence of symptoms of anxiety disorder and depressive disorder having increased more than 3-fold in the United States during the COVID-19 pandemic [45,46]. Furthermore, people with few social and economic resources had high likelihood of exhibiting depression symptoms during this time [46]. Digital tools are therefore increasingly relevant in the era of COVID-19, owing to increased use of telehealth services to facilitate access to care [47]. In addition, low-cost interventions, such as digital tools, could provide increased monitoring and improved services to at-risk populations [15,48].

Moreover, future research methodologies, analysis protocols, and publications should provide a more explicit account of the impact of people’s social intersectionality on their perceptions about remote MDD monitoring. The imperative for this is heightened by the increasing attention that reimbursement entities are paying to the way any given intervention can help minimize the negative effects that social determinants have on treatment outcomes [49].

### Conclusions

Ongoing communication with patients with MDD and their care teams (cocreation) provided an opportunity for continued reassessment and developmental iteration of *Pathway Platform*. These insights included the need for rapid communication of updated and current patient data with the care team, integration of the app into the MDD care pathway via the EHR, and education of the care team about the interpretation and use of these data. This cocreation model using qualitative research findings has resulted in fast iterations and optimized system performance and will allow for eventual sustainability outside the research environment. Future development of *Pathway Platform* will continue, consistent with the evolving needs of people living with MDD and their care teams.

## Appendix

Multimedia Appendix 1 The treatment journey for patients with major depressive disorder (MDD).

Multimedia Appendix 2 Screenshot of the *Pathway app* and a sample report from the pilot study.

Multimedia Appendix 3 Details of health care provider sample in stage-2 interviews.

Multimedia Appendix 4 Design of *Pathway Platform*.

Multimedia Appendix 5 User-centered design approach.

## Abbreviations

AAH: Advocate Aurora Health
EHR: electronic health record
HCP: health care provider
MBC: measurement-based care
MDD: major depressive disorder
PHQ-9: 9-item Patient Health Questionnaire
PRO: patient-reported outcome
SDM: shared decision-making

## Data Availability

The data sets generated and analyzed during this study are available from the corresponding author upon reasonable request.
