# Supplementary figures and images for: User-Centered Design of a Digitally Enabled Care Pathway in a Large Health System: Qualitative Interview Study
Source: JMIR Hum Factors. 2023 Jul 26;10:e42768. doi: 10.2196/42768 (PMC10413250; doi:10.2196/42768)

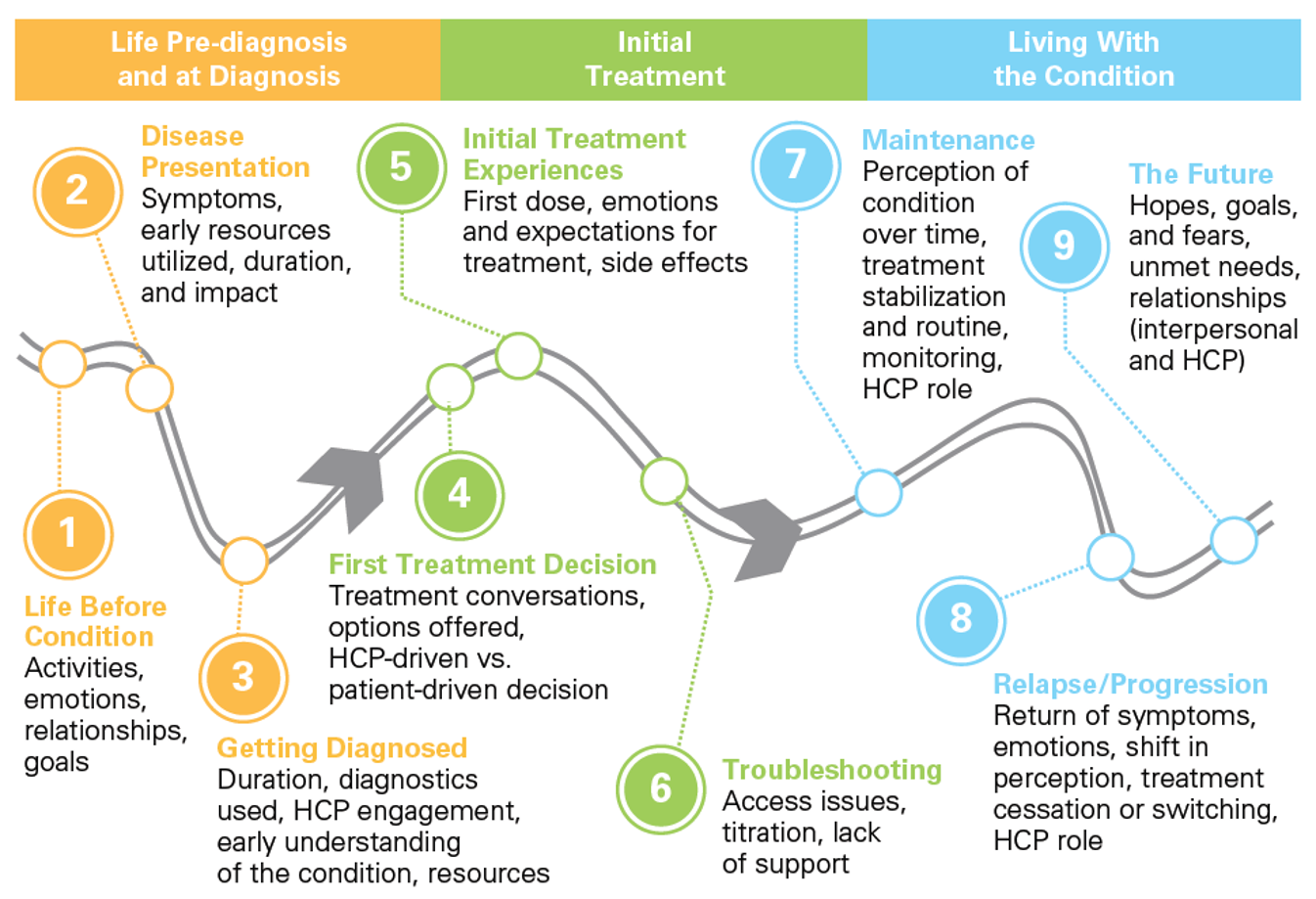

Supplement: Multimedia Appendix 1 [file humanfactors_v10i1e42768_app1.docx]

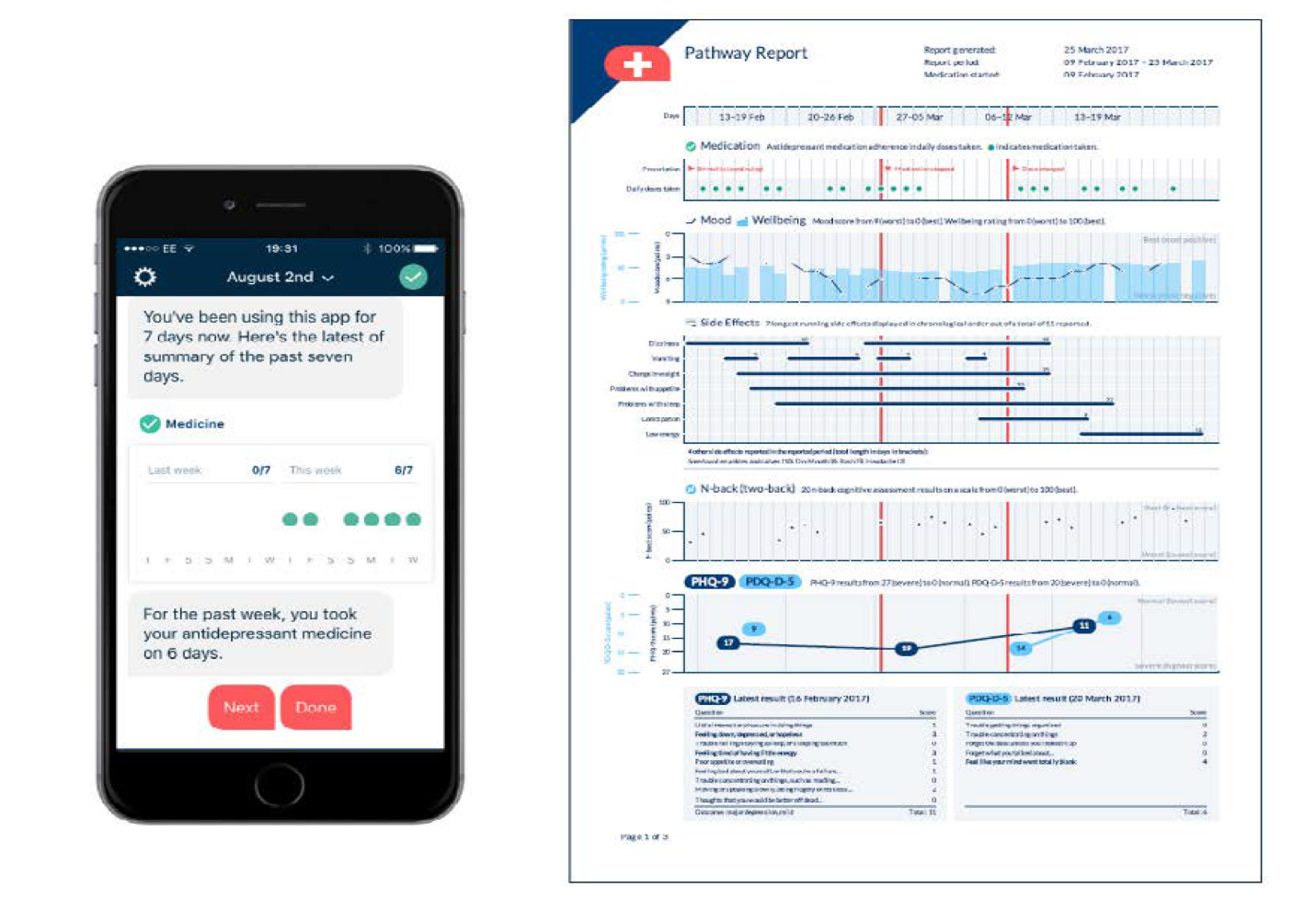

Supplement: Multimedia Appendix 2 [file humanfactors_v10i1e42768_app2.docx]

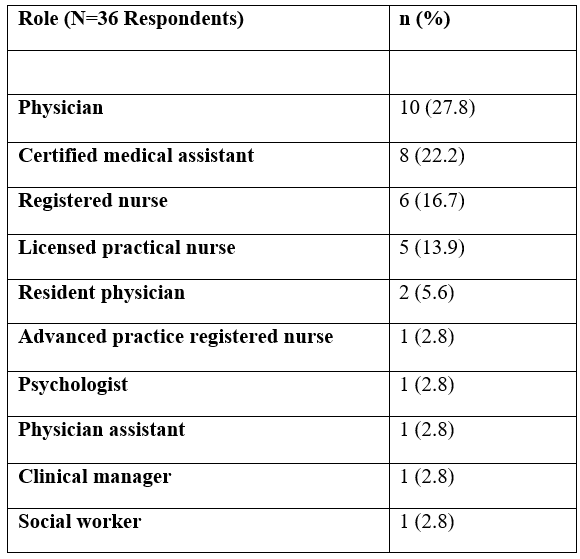

Supplement: Multimedia Appendix 3 [file humanfactors_v10i1e42768_app3.docx]

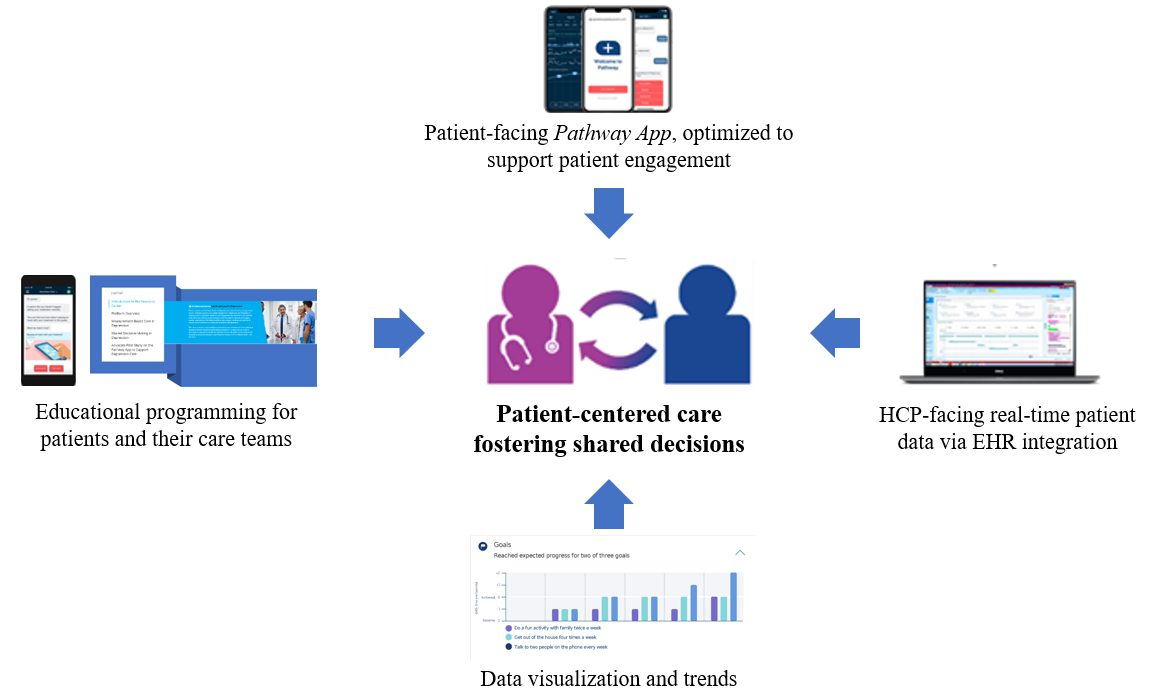

Supplement: Multimedia Appendix 4 [file humanfactors_v10i1e42768_app4.docx]

**
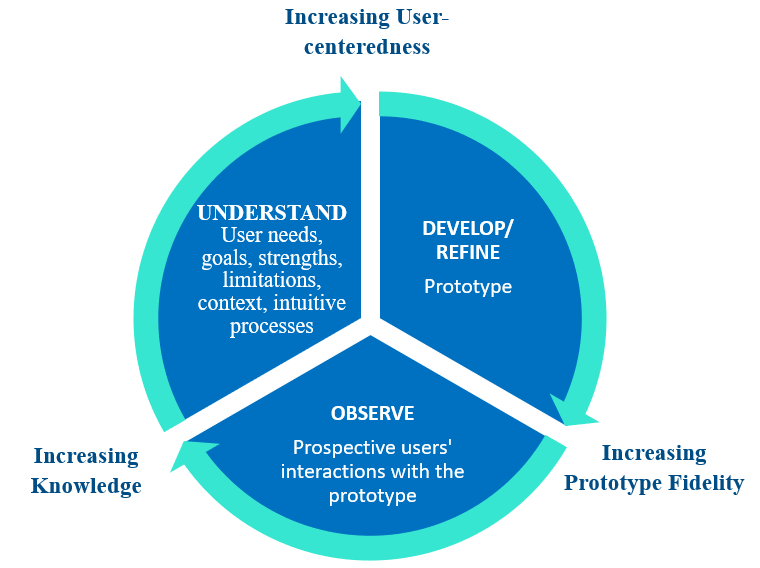
**

Supplement: Multimedia Appendix 5 [file humanfactors_v10i1e42768_app5.docx]
